# EDT-MCFEF: a multi-channel feature fusion model for emergency department triage of medical texts

**DOI:** 10.3389/fpubh.2025.1591491

**Published:** 2025-06-18

**Authors:** Tao Lin, Shiming Yi

**Affiliations:** School of Computer Science and Information Engineering, Shanghai Institute of Technology, Fengxian District, Shanghai, China

**Keywords:** artificial intelligence, intelligent medical systems, emergency department triage, natural language processing, classification

## Abstract

**Introduction:**

Triage is a pivotal function within the operational framework of an emergency department, as it directly influences patient outcomes and hospital efficiency. However, traditional triage methods frequently depend on human judgment, which is susceptible to high subjectivity and low efficiency.

**Methods:**

To address these issues, this paper presents a novel emergency department triage algorithm. The proposed EDT-MCFEF (Emergency Triage Algorithm Based on Multi-Channel Feature Extraction and Fusion) addresses numerous shortcomings of conventional triage methodologies. The model employs a hybrid masking approach and RoBERTa (Robustly Optimized BERT Approach) to facilitate feature enhancement and word vector processing of text. Moreover, the model employs a convolutional neural network (CNN) and a multi-headed attention (MHA) mechanism to extract text features from multiple channels, effectively capturing both local and global features. Furthermore, this paper introduces a multi-channel feature fusion method, which integrates local and global features and achieves comprehensive learning and optimization of feature information through dynamic weight adjustment.

**Results and discussion:**

The objective of this model is to enhance the accuracy and efficiency of emergency department triage, thereby providing scientific and technological support to the emergency department. In this paper, two medical text datasets are employed for experimental validation: a self-built emergency department triage dataset and a medical literature abstract dataset. The emergency department triage dataset consists of 28,000 English-annotated samples from 11 clinical departments, while the medical literature abstract dataset is a publicly available dataset (https://huggingface.co/datasets/123rc/medical_text). The experimental findings demonstrate that the proposed model exhibits superior accuracy to seven benchmark models utilized in this study on both medical text datasets, indicating its efficacy in handling imbalanced datasets. This suggests enhanced generalization and robustness. In addition to its strong classification ability, the model exhibits favorable interpretability through its multi-channel design, and the hybrid masking strategy supports data minimization and privacy protection, aligning with ethical AI principles. This approach holds promise for integration into clinical decision support systems for improved triage accuracy. The models and the self-built dataset presented in this paper are available at https://github.com/Yiii-master/EDT-MCFEF.

## 1 Introduction

The emergency department represents the central operational unit within a hospital, tasked with responding to acute medical conditions and urgent medical situations. As the population ages and the prevalence of chronic diseases increases, the number of patients in emergency departments continues to rise, creating unprecedented challenges for triage systems. The reliance on subjective experience and fixed rules in traditional triage processes makes the system susceptible to miscalculations in high-pressure environments, which can affect the timeliness of treatment. The major challenges currently facing emergency triage systems include the efficient processing and analysis of large amounts of patient medical record data, the accurate extraction of key information from unstructured text to aid decision-making, and the inadequacy of the existing triage process in terms of personalization and flexibility, which makes it difficult to meet the individualized treatment of different patient needs.

The advent of artificial intelligence technology has led to a paradigm shift in the field of research, with the focus now shifting toward the development of automated triage systems. Natural language processing (NLP) techniques, with a particular emphasis on deep learning-based models, have demonstrated considerable promise for implementation in the domain of medical text analysis. Nevertheless, extant methodologies continue to exhibit the following deficiencies when confronted with emergency triage tasks:

1. Insufficient feature extraction: traditional methods such as bag-of-words models and static word vectors make it difficult to capture complex semantic information in medical texts (1).
2. Global and local feature imbalance: existing models demonstrate limited efficacy in capturing global dependencies and local features (2). Typically, these models employ a fixed feature fusion strategy that cannot dynamically adjust feature weights according to task requirements.
3. Data noise interference (3): medical texts are frequently characterized by an abundance of extraneous information and terminology, which can compromise the efficacy of classification models.

In order to address the aforementioned challenges, and to develop a deep learning framework that is robust, interpretable, and privacy-preserving for automated triage classification in medical text scenarios, this paper proposes a novel multi-channel feature extraction emergency department triage model (EDT-MCFEF), which employs a feature enhancement method that combines a hybrid mask and RoBERTa. The model utilizes a multi-channel feature fusion approach, dynamically adjusting the learning weights of the features on the multi-channel features to optimize the model's learning process among the different channel features. The aforementioned strategies facilitate the comprehensive extraction and learning of text features by the model.

The main contributions of this paper are as follows:

- This paper employs a model training method based on the hybrid mask method. In regard to the data input to the model, this paper divides it into two parts according to a specific ratio and enhances it with an n-gram mask and a filler mask, respectively. The two parts of the processed data are then combined. This method not only enhances the differentiation of the data, but also reduces the model's dependence on common but non-critical features, thus providing a richer and more effective feature representation for deep learning model training.
- The feature extraction layer of the model presented in this paper employs an integrated approach combining the Multi-Head Attention Mechanism (MHA) with the Convolutional Neural Network (CNN). The MHA is utilized to extract global feature information from the text, while the CNN is employed to extract features from local context. The multi-channel feature extraction strategy is capable of comprehensively capturing both global and local features of the text, particularly in the context of emergency department triage. By synthesizing the overall trend with specific details such as symptom keywords, the model is able to improve its accuracy and robustness.
- This paper introduces a multi-channel feature integration strategy at the integration learning layer of the model. This strategy enables the adaptive learning of the relative importance of each feature in accordance with the task requirements, through the dynamic adjustment of feature weights. The flexible fusion mechanism allows for the retention of key information to the greatest extent possible, while simultaneously optimizing the integrated use of key features. The results of the experiments demonstrate that this approach enhances the accuracy and robustness of the triage system.

## 2 Related works

Text categorization is the process of automatically assigning a text to a predefined category or label. The emergency department triage task is the process of classifying medical texts pertaining to emergency patients into the appropriate departments. The conventional machine learning methodologies, which typically employ bag-of-words (4) models, entail a static word vector processing methodology. This is achieved through the construction of dictionaries and the utilization of support vector machines (SVM) (5) for classification. These processes and models are overly reliant on human involvement in feature engineering, and the models exhibit suboptimal generalization capabilities.

Deep learning does not necessitate the construction of intricate feature engineering models and is therefore able to automatically extract meaningful latent information and discriminative features from a vast quantity of data. Consequently, it has become a prevalent methodology for natural language processing tasks, including text categorization. Improved text classification models based on existing neural network models, such as TextCNN (text convolutional neural network) (6) and RNN (recurrent neural network) (7) [e.g., LSTM (8) and BiLSTM (9)], are rapidly deployed in text classification tasks. The Very Deep Convolutional Neural Network (VDCNN) (10) enhances the efficacy of text categorization by augmenting the network depth and integrating the Residual Connections method to mitigate gradient vanishing and expedite model training. Researchers (11) introduced the C-LSTM model in 2015, which combines the features of CNN and LSTM to enable the model to learn local features as well as capture global temporal features.

Nevertheless, network architectures such as CNN and RNN continue to exhibit limitations in their ability to capture semantic relations between words and remain constrained by the use of static word vector processing through bag-of-words models. In response to these challenges, the Transformer (12) model was proposed in 2017, which employs an encoder-decoder structure to dynamically obtain the word vector representation of text. This approach enables the model to achieve feature extraction capability and large-scale data training efficiency through a robust parallel computing framework. A number of pre-training models based on the Transformer architecture, including GPT (Generative Pre-trained Transformer) (13) and BERT (Bidirectional Encoder Representations from Transformers) (14), have also demonstrated remarkable performance. The optimized BERT-based model RoBERTa (15) was developed in 2019 by utilizing a larger dataset and more training time. This model employed the elimination of the Neighborhood Sentence Prediction (NSP) task during training and incorporated more data for training, thereby enhancing the model's robustness. Subsequently, Some researchers (16) enhanced the RoBERTa model through the implementation of an LSTM-CNN methodology, thereby optimizing the results on the sentiment classification task. Similarly, Qian Wang and other researchers (17) enhanced the pre-training model RoBERTa by incorporating an additional attention pooling layer to reduce the feature scale and complete the classification output subsequent to feature extraction using RCNN (RNN-CNN). The model was validated by applying it to the news text classification task. Following the introduction of the RoBERTa model, Sun and other researchers (18) devised ERNIE, a model tailored to Chinese NLP tasks, by analyzing the characteristics of Chinese text and integrating a greater quantity of external knowledge in 21. In 2023, the LLaMA (19) model, which is also based on the Transformer architecture, was proposed. It employs the latest optimization techniques, including pre-normalization and the SwiGLU activation function, to enhance training stability and performance.

In the context of deep learning-based emergency department triage, some researchers (20) have proposed an approach to sentence-level clinical text categorization that employs convolutional neural networks (CNNs) to represent the intricate nuances of sentences. Some scholars (21) have developed a framework that integrated CNNs and Deep Boltzmann Machines (DBMs) to facilitate the representation of documents for semantic indexing of biomedical literature. In this approach, CNNs are utilized to extract contextual local information, whereas the DBM is employed to integrate global features. Some researchers (22) employed a Triage Priority (TP) approach to label diagnostic combinations in medical records for processing medical texts and incorporated a composite loss function based on cost-sensitive learning (CSL) into the BERT architecture. This learning-based composite loss function addressed the issue of data imbalance, while Zhang (23) superimposed an RNN on the original BERT architecture and utilized the model to classify the medical literature on cancer. In recent years, MedFound (24) was proposed and used to optimize clinical text representation through pre-training in the medical domain, while Toma et al.'s (25) Clinical Camel was fine-tuned based on LLaMA-2 to propose “conversational knowledge encoding” for synthesizing conversational data to improve the performance of biomedical tasks. However, these models are not optimized for local-global feature dynamic fusion of spoken text in emergency triage, and EDT-MCFEF addresses this gap through hybrid masking with a multi-channel dynamic weighting mechanism.

## 3 Our method: EDT-MCFEF

**Figure 1:**
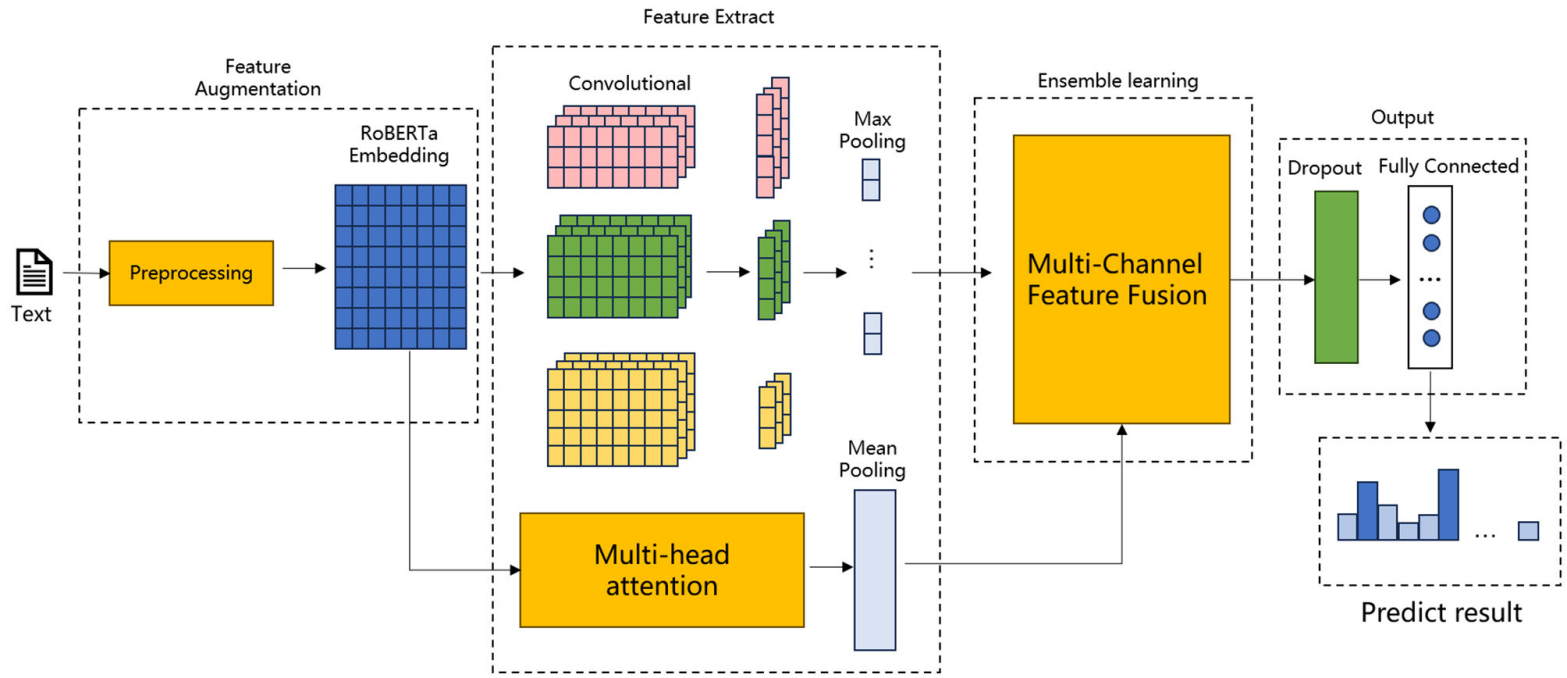
Structure of EDT-MCFEF.

As illustrated in Figure 1, the model's fundamental structure is based on the RoBERTa model, which is integrated with an additional model, MHA-CNN, for feature extraction purposes. The meticulous design of the model proposed in this paper facilitates the comprehension and analysis of intricate textual data, particularly the precise and expeditious extraction of pivotal features in emergency department triage operations.

**Figure 2:**
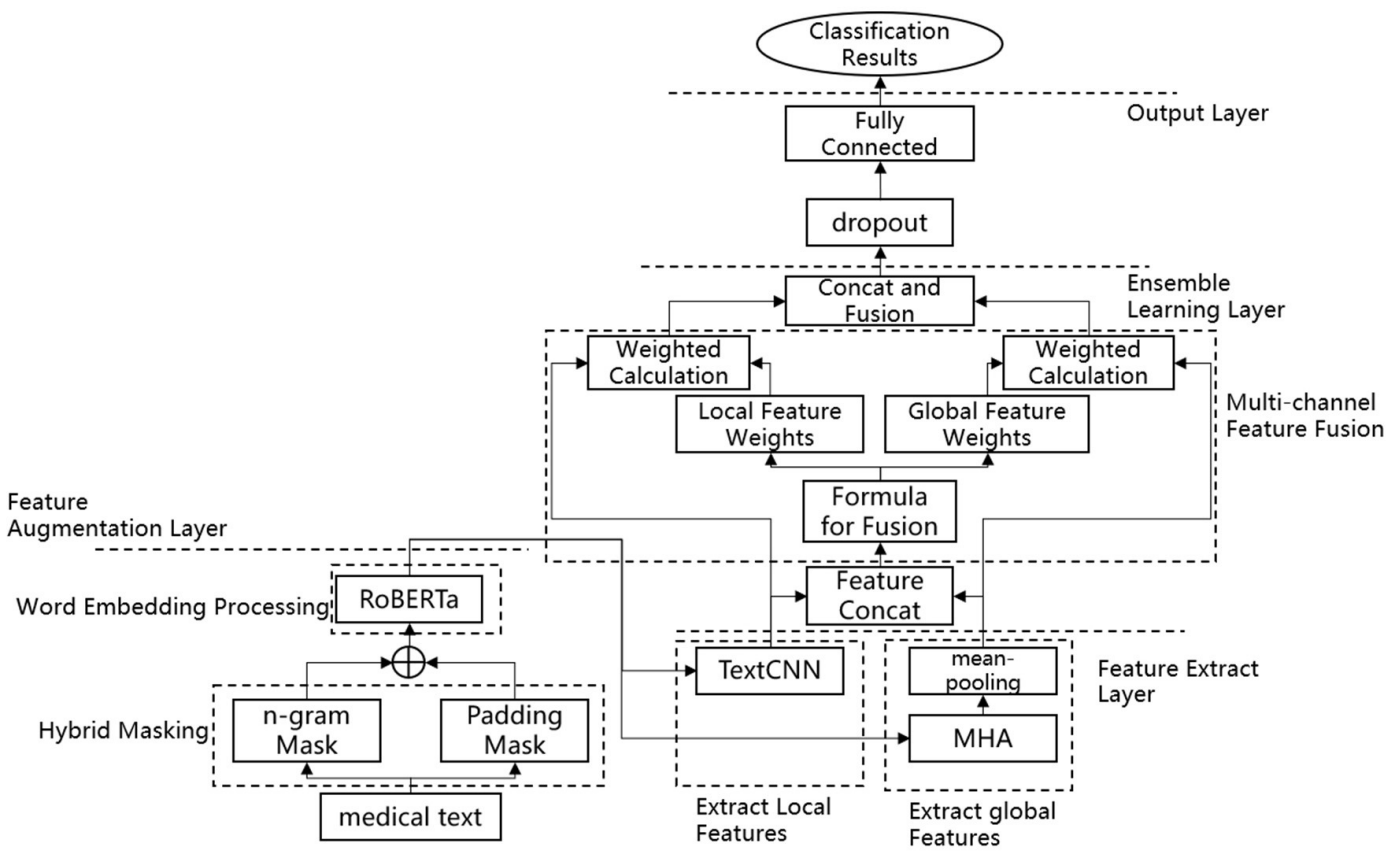
Flowchart of the EDT-MCFEF model.

Figures 1, 2 illustrates the proposed model, which incorporates a feature augmentation layer, a feature extraction layer, an ensemble learning layer, and an output layer. By employing a multi-level processing approach, the EDT-MCFEF model is able to achieve feature augmentation through the use of a hybrid mask and a RoBERTa model. Subsequently, it learns and fuses an ensemble learning model, namely MHA-CNN, with the objective of strengthening the expression of semantic information. This ultimately enables the model to perform high-precision text categorization in the context of emergency department triage.

- Feature augmentation layer: In this paper, a hybrid masking strategy combining n-gram mask and padding mask is adopted for data preprocessing, which encourages the model to avoid over-reliance on frequently occurring features that may not be discriminative, and enables the model to better understand valid contextual information, improving training efficiency and classification accuracy. After these two masking steps, the text sequences are fed into the RoBERTa model for training, and RoBERTa achieves further feature augmentation by generating context-dependent word vector representations through a bidirectional encoder structure, which provides rich information for the subsequent feature extraction phase.
- Feature extraction layer: the feature extraction layer is a component of the overall system that is responsible for the extraction of features from the input data. In this paper, we adopt the MHA-CNN Ensemble model, which has the objective of simultaneously extracting multi-scale features in text, including global dependencies and local features. Among these, the MHA extracts the global semantic information of the text by computing multiple attention heads in parallel, thereby capturing the long-distance dependencies between the parts of the text. In contrast, the CNN focuses on the extraction of n-gram features from a local context. This layer enables the model to synthesize the global dependencies and local semantic features of the text, thereby augmenting the model's capacity to comprehend complex textual information. In the context of emergency department triage, the global semantic information of the text, such as the overall trend of the medical record description, and the local features, such as the keywords of the specific symptom, are of equal importance. The model proposed in this paper improves the accuracy and robustness of the model by employing a dual feature extraction strategy.
- Ensemble learning layer: this paper introduces a multi-scale feature ensemble strategy to achieve the effective fusion of global and local features. The essence of this strategy lies in the fact that by employing ensemble learning to combine the outputs of multiple feature sources and dynamically adjusting the weights of each feature, the model is able to adaptively learn the relative importance of each feature in accordance with the varying requirements of the task. By designing an accurate fusion formula, the model is able to obtain the dynamic weights of global and local features from training. Prior to fusion, the weights and their respective corresponding module outputs are weighted, which allows the model to dynamically learn the feature information of the two channels during the training process and ensures that the key information is retained during feature fusion.
- Output layer: the output layer comprises a dropout (26) layer and a fully connected layer. The final comprehensive semantic features are fed into the dropout layer with the objective of reducing the neural network complexity and preventing overfitting, whereby some neurons are randomly discarded. Subsequent to processing in the fully connected layer, the model maps the final output to the appropriate categories, thereby obtaining the emergency department triage results.

### 3.1 Feature augmentation layer

#### 3.1.1 Hybrid masking

In NLP tasks, the performance of a model is frequently influenced by the quality of the input data, particularly in the presence of substantial quantities of noise or invalid information. For this reason, this paper employs two masking techniques, namely n-gram masks and padding masks, and uses them in combination as a data enhancement strategy. This approach enables the model to focus on more informative features while effectively reducing the interference of irrelevant and redundant content, thereby enhancing the robustness and generalization ability of the model. Hybrid masking involves the division of the input data into two parts, 70% is allocated for n-gram masking, and 30% is designated for padding masking. This approach guarantees that the model will benefit from two distinct masking strategies. Tables 1, 2 below illustrate the types of n-gram masks and padding masks that can be employed. In these tables, “1” indicates that the computer has recognized the sub-word, while “0” indicates that the computer has failed to recognize the sub-word. At the same time, the hybrid masking mechanism can protect potentially sensitive features—such as patient information that is irrelevant to the algorithmic model—thereby reducing privacy risks in artificial intelligence applications and reflecting a design philosophy aligned with AI ethics principles.

**Table 1:** Examples of n-gram masks.

**Text**	**2-gram**		**3-gram**	
	Mask text	Computer recognition	Mask text	Computer recognition
I have a headache	I have ##	1,100	I ###	1,000

**Table 2:** Examples of padding masks.

**Text**	**Pad size = 3**		**Pad size = 5**	
	Mask text	Computer recognition	Mask text	Computer recognition
I have a headache	I have a #	1,110	I have a headache #	11,110

#### 3.1.2 RoBERTa

**Figure 3:**
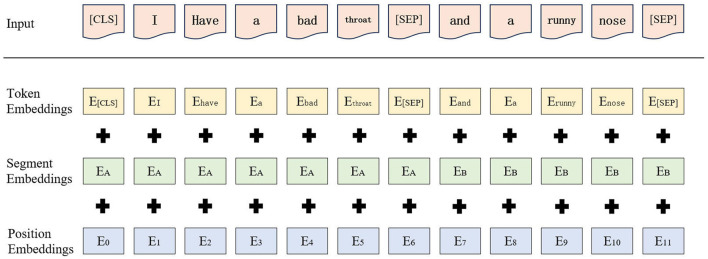
Embedding layer processing of RoBERTa.

Given RoBERTa's demonstrated proficiency in feature representation, this study employs the model to enhance the data quality of emergency medical texts. RoBERTa performs a basic segmentation of the input sequence, whereby the text is first split into individual sub-words, which then form the input sequence for the model. In addition, RoBERTa introduces two distinct types of tokens: [CLS] and [SEP]. The former is utilized to demarcate the onset of the input sequence, while the latter serves to delineate the segmentation between sentences, thereby enabling the model to discern the boundaries of disparate sentences. In RoBERTa, the word embedding of each sub-word is comprised of three distinct sub-layers: token embedding(TE), segment embedding (SE), and position embedding (PE). The sum of the outputs from these three sub-layers constitutes the word vector that is inputted to the encoding layer, *E*. The embedding layer of RoBERTa is illustrated in Figure 3.

The process of obtaining word vectors through RoBERTa training is as follows: first, the input sequence is passed into the RoBERTa model. In the embedding layer of the model, each sub-word in the input sequence is mapped to a high-dimensional word vector, denoted as *E*_*i*_ (*i* = 1, 2, 3, ..., *n*), i.e., as shown in Equation 1.


(1) $$\begin{array}{l} E_{i}=TE_{i}+SE_{i}+PE_{i} \end{array}$$

Subsequently, the word vectors are fed into multiple coding layers, where they are processed by a multi-head self-attention mechanism and a feed-forward neural network in order to progressively update their representations. The output representation of each layer is not merely the consequence of the current layer's computation; it also serves as part of the next layer's input, thereby facilitating the transfer and transformation of information in each layer of the model. In essence, the output of RoBERTa, which represents the sequence *T* = (*T*_1_, *T*_2_, ..., *T*_*n*_), is a combination of the outputs of all layers. These are typically accumulated or fused with the outputs of all hidden layers (of hidden size).

The employment of a hybrid masking approach in conjunction with RoBERTa for feature augmentation offers potential advantages within the domain of emergency department triage. First, the integration of the n-gram mask and filler mask in the hybrid masking strategy is expected to enhance the differentiation of data and reduce the model's reliance on common but non-critical features. This approach is anticipated to improve the model's ability to capture key information in medical texts. Secondly, RoBERTa, as a pre-trained model based on Transformer, is capable of dynamically generating contextually relevant word vector representations, thereby facilitating a more nuanced comprehension of the intricate semantic relationships present in medical texts. The integration of these two approaches enables the model to extract rich features from a substantial corpus of unstructured medical texts and to adapt to a variety of text scenarios in emergency department triage tasks. This, in turn, leads to a substantial enhancement in the accuracy and robustness of classification.

### 3.2 Feature extraction layer: multi-channel feature extraction (MHA-CNN)

#### 3.2.1 MHA

The Self-Attention (SA) mechanism (27) is a mechanism for weighting attention to information at different locations in an input sequence. This mechanism has the advantage of enabling the model to focus attention on the parts of the input sequence that are relevant to a particular task, thus improving the model's performance.


(2) $$\begin{array}{l} Attention\left(Q,K,V\right)=Softmax\left(\frac{QK^{T}}{\sqrt{d^{K}}}\right)V. \end{array}$$

In the basic Equation 2 of the self-attention mechanism, the values of *Q*, *K*, and *V* are all obtained by dot-product computation of the input embedding representation *X* and the weight matrices *W*^*Q*^, *W*^*K*^, and *W*^*V*^. The result of *QK*^*T*^ reflects the similarity between *Q* and *K*, and the result is multiplied by a scaling factor $\frac{1}{\sqrt{d^{K}}}$ to avoid the problem of vanishing gradient. The result of $\frac{QK^{T}}{\sqrt{d^{K}}}$ is normalized and computed using the SoftMax function, and the attentional output is obtained by multiplying the normalized attentional weights with the corresponding *V*.

MHA represents a pivotal element of the Transformer model, which extends the conventional attention mechanism by enabling the model to concurrently attend to disparate aspects of the input in varying representation subspaces. This approach enables the model to simultaneously learn diverse features in different representation subspaces.


(3) $$\begin{array}{l} Z=MultiHead\left(Q,K,V\right)\\ =Concat\left(head_{0},head_{1},head_{2},...,head_{h}\right)W^{O}. \end{array}$$

(4) $$\begin{array}{l} head_{i}=Attention\left(QW_{i}^{Q},KW_{i}^{K},VW_{i}^{V}\right). \end{array}$$

Equations 3, 4 show the computational part of the MHA, *head*_*i*_ denotes the output of the i-th attention head, *W*^*O*^ is the linear transformation matrix used to splice the outputs of all the attention heads and then perform a linear transformation to get the sequence *Z* that can be input to the fully connected layer for classification, $W_{i}^{Q}$, $W_{i}^{K}$, and $W_{i}^{V}$ are linear transformation matrices used by each head to get *Q*, *K*, and *V*.

#### 3.2.2 TextCNN

**Figure 4:**
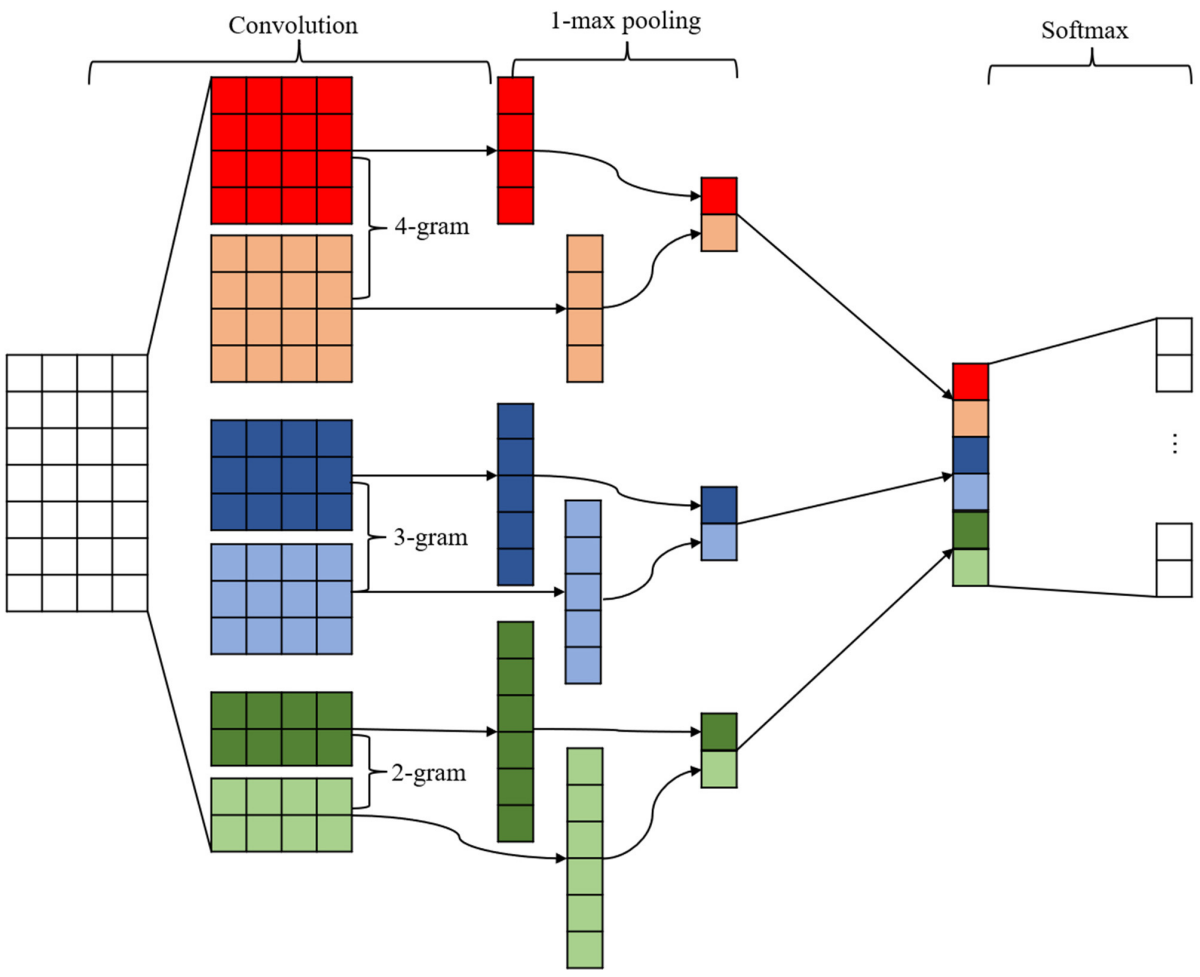
Structure of TextCNN model.

We have improved the input layer of the CNN so that the neural network can be applied on text feature extraction. The network primarily comprises a convolutional layer, a pooling layer, a splicing layer, and a fully-connected layer. TextCNN initially captures the local features of the input sequence through convolutional operations, with the width of the convolutional kernel typically corresponding to the size of the n-gram and the height corresponding to the dimensionality of the word embeddings. The model typically employs a plurality of convolutional kernels for the purpose of capturing n-gram features of varying dimensions. Subsequently, TextCNN reduces the feature dimensionality through a maximum pooling layer, thereby extracting the salient information present within the text. All the pooled features are then spliced or concatenated into a vector, which is subsequently input to the fully connected layer for text classification. The advantage of TextCNN is that it can effectively and rapidly capture local features in short texts, and its structure is relatively simple and straightforward to comprehend and implement. Figure 4 illustrates the fundamental structure of TextCNN.

### 3.3 Ensemble learning layer: multi-channel feature fusion strategy

**Figure 5:**
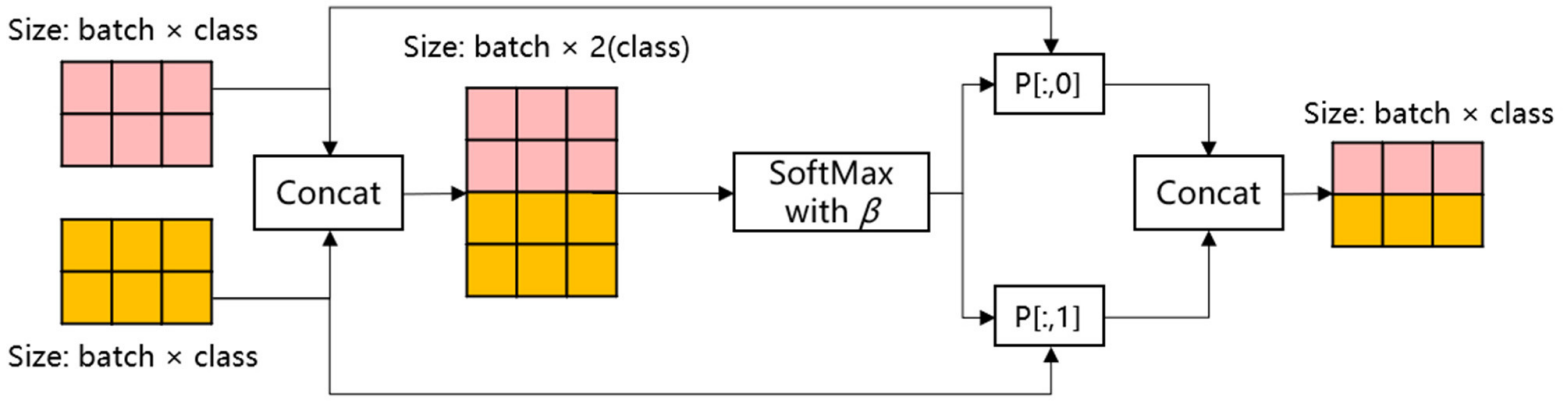
Process of multi-channel feature fusion strategy.

In order to enhance the model's performance and generalization ability by effectively integrating diverse information from disparate feature sources, this paper employs a multi-channel feature fusion approach to ensemble learning, as illustrated in Figure 5.

In this ensemble strategy, the two classes of outputs of the integrated model MHA-CNN in the feature extract layer are initially spliced in accordance with the specifications set forth in Equation 5:


(5) $$\begin{array}{l} C=Concat\left(Out_{MHA},Out_{CNN}\right) \end{array}$$

The location of the aforementioned item is as follows: In this context, *Out*_*MHA*_ and *Out*_*CNN*_ represent the respective outputs of MHA and CNN in the feature extraction layer, specifically global and local features, respectively. The term “*Concat*” denotes the concatenation operation, and *C* is the result of this operation.

Subsequently, the SoftMax function was employed with a single temperature parameter β as the output weight for the fusion formula for multi-channel feature fusion, as detailed below:


(6) $$\begin{array}{l} P\left(C_{i},\beta \right)=\frac{e^{C_{i}/\beta }}{\Sigma_{j=1}^{N}e^{C_{j}/\beta }} \end{array}$$

The location of the aforementioned item is as follows: In this context, *C*_*i*_ represents the i-th element of the input vector *C*. The term $e^{C_{i}}$ ensures that the resulting output is positive. The denominator of the formula is the exponential sum of all the elements in the input vector. This ensures that the output values are normalized, with the result that the sum of all the outputs is 1. The symbol *P* denotes the probability distribution of the input vector. The shape of *P* is (N, 2). This means that for each of the N samples, the probability distribution *P* contains two weights. These are the weights of global features and local features, respectively.

In particular, a temperature parameter β is introduced to participate in the calculation of SoftMax, which controls the smoothness of the distribution of the output weights. When β is small, the difference between the inputs in the SoftMax function is amplified, resulting in a high probability of a certain category and a bias toward that category. Conversely, when β is large, the difference between the inputs in the SoftMax function is narrowed, and the model's prediction is more uncertain. The feature ensemble strategy may be adapted to suit different tasks by adjusting the size of β.

Once the probability distribution *P* has been obtained, the feature representation *H* is then output after feature ensemble by means of the equations provided in Equations 7–9.


(7) $$\begin{array}{l} \tilde{O}ut_{MHA}=P\left[;,0\right]\cdot Out_{MHA} \end{array}$$

(8) $$\begin{array}{l} \tilde{O}ut_{CNN}=P\left[;,1\right]\cdot Out_{CNN} \end{array}$$

(9) $$\begin{array}{l} H=\tilde{O}ut_{MHA}+\tilde{O}ut_{CNN} \end{array}$$

In this context, *P*[:, 0] is indicative of the weights associated with the MHA, while *P*[:, 1] is representative of the weights pertaining to the CNN. The respective outputs of MHA and CNN in the feature extract layer are weighted and fused with the corresponding weights, thereby obtaining the comprehensive semantic feature *H*.

Existing methods achieve this by straightforwardly splicing MHA and CNN features, yielding a model that grapples with differentiating primary from secondary information in intricate text. The temperature parameter-controlled fusion strategy (Equation 6) proposed in this paper dynamically adjusts the weights according to the input text. When the patient description contains explicit keywords such as “chest pain,” the CNN local feature weights increase (β decreases). Conversely, when relying on contextual inference, such as “relieved after resting,” MHA global feature weights prevail (β increases).

The method maximizes the effectiveness of the features through a dynamic fusion strategy that flexibly weighs the features extracted by the MHA and CNN, and is able to adaptively adjust the feature weights according to the characteristics of the input text. The MHA excels in capturing the global dependencies in the medical text and comprehensively understands the contextual semantic information, whereas the CNN focuses on the extraction of the local features to accurately recognize medical terms and phrase-level patterns. The dynamic fusion strategy is expected to address the limitations of conventional static fusion methods in adapting to diverse text scenarios. In addition, it is anticipated to enhance the model's capacity to process complex medical texts. This multi-channel feature fusion method is expected to improve the accuracy and robustness of emergency department triage, providing a smarter and more flexible solution for classifying emergency medical records and reducing the reliance on manual feature engineering. Consequently, it is anticipated to improve the practicality and efficiency of the model.

### 3.4 Output layer

**Figure 6:**
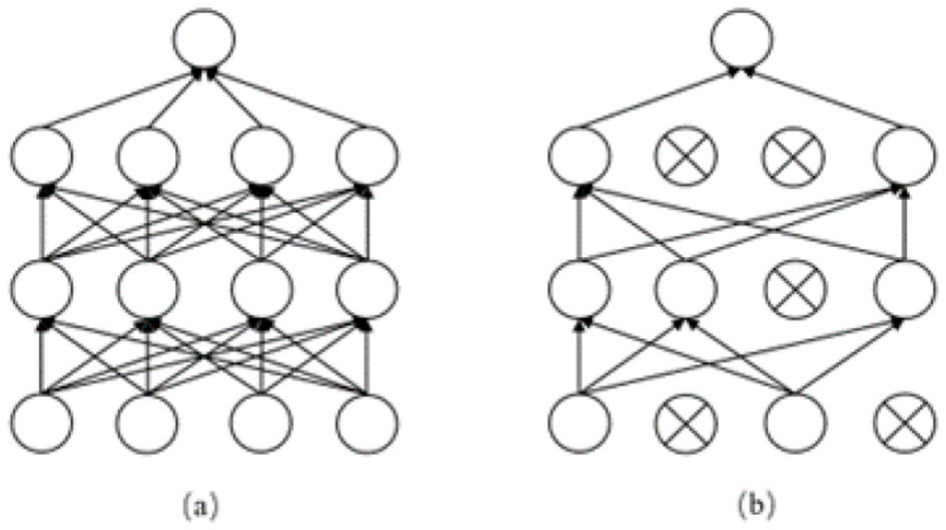
**(a)** shows the neural propagation graph before adding dropout, **(b)** shows the neural propagation graph after adding dropout.

Subsequently, the output *H* of the ensemble learning layer is conveyed to the output layer, where it is initially subjected to processing through the dropout layer. Dropout is a regularization technique employed in the training of neural networks with the objective of reducing overfitting. As illustrated in Figure 6, the training process involves the random deactivation of certain neurons, thereby preventing the network from becoming overly reliant on a limited set of neurons. This approach enhances the model's ability to generalize and perform well on new data. Subsequently, the output *H* from the ensemble learning layer is mapped to the corresponding category, namely the result of emergency department triage. The workflow of the output layer is illustrated in Equations 10, 11.


(10) $$\begin{array}{l} H_{dropout}=Dropout\left(H,p\right) \end{array}$$

(11) $$\begin{array}{l} y=SoftMax\left(W\cdot H_{dropout}+b\right) \end{array}$$

Equation 10 illustrates the dropout operation, where *H* denotes the output of the integrated learning layer, *p* signifies the probability of dropout, and *H*_*dropout*_ represents the output following dropout processing. Equation 11 subsequently delineates the classification mapping process, wherein *W* denotes the weight matrix, *b* signifies the bias vector, and *y* represents the ultimate classification outcome (emergency department triage result).

## 4 Experiments and analysis of results

### 4.1 Dataset and experimental environment

**Figure 7:**
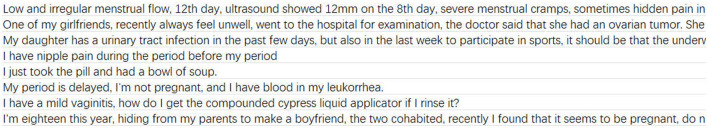
Some examples of self-built medical datasets.

**Figure 8:**
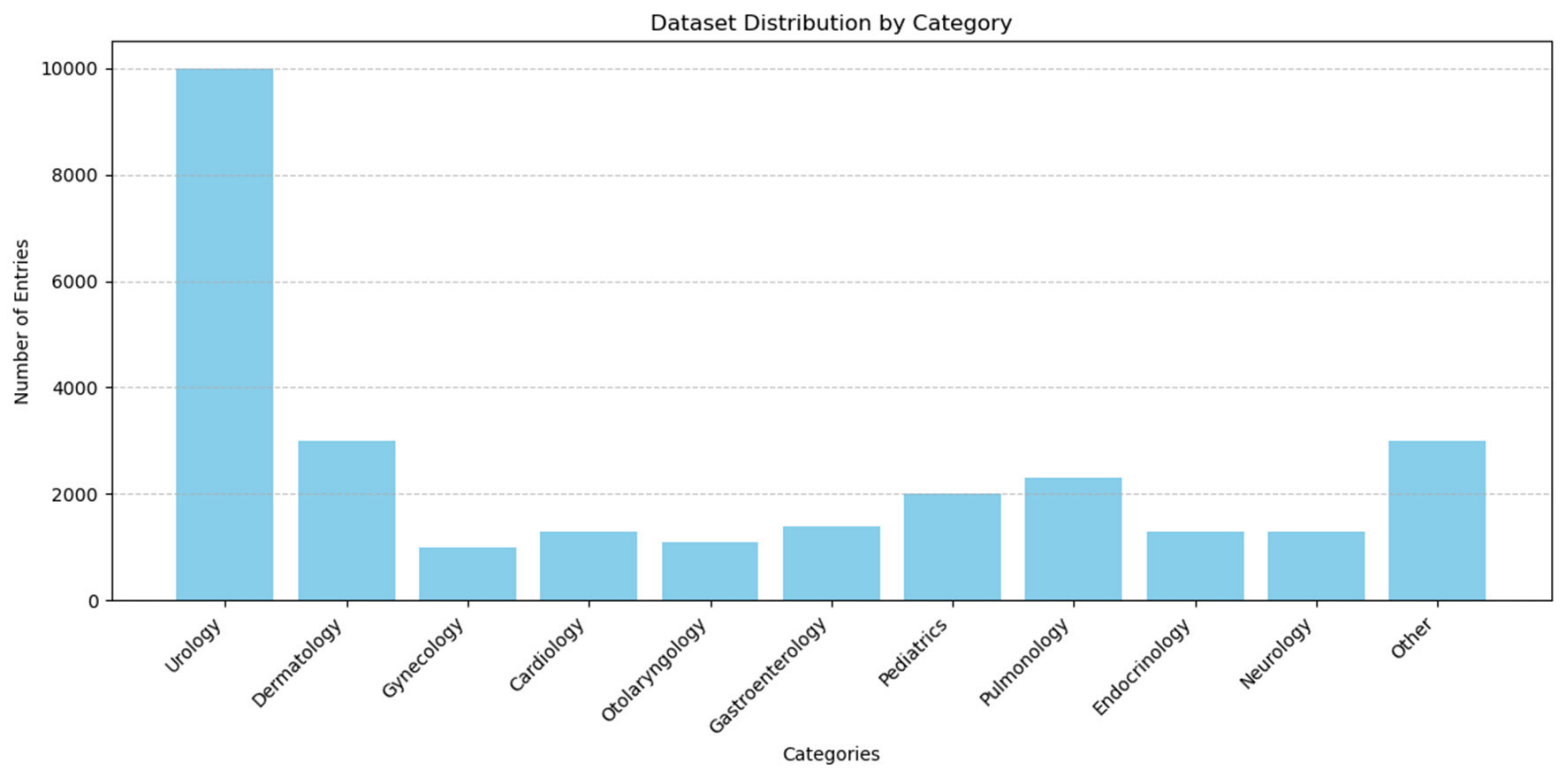
Statistical results of the self-built medical dataset.

In order to ascertain the efficacy of the model presented in this paper in achieving the classification of medical texts and thus emergency department triage, a self-collected medical dataset was employed for the purpose of evaluating the emergency department triage algorithm. The dataset was primarily collected through the administration of an anonymous questionnaire and the use of simulated patient descriptions. The dataset consisted of 28000 entries, which were divided into training, validation, and testing sets in a ratio of 8:1:1. The departmental categories included Urology (10,000 entries), dermatology (3,000 entries), and gynecology (1,000 entries). The remaining entries were allocated to the following categories: Cardiology (1,300 entries), otolaryngology (1,100 entries), gastroenterology (1,400 entries), pediatrics (2,000 entries), pulmonology (2,300 entries), endocrinology (1,300 entries), neurology (1,300 entries), and “other” (3,000 entries). The mean length of the texts was 92 words, Figures 7, 8 present several examples of self-built datasets, along with the statistical results obtained from their analysis. The class distribution in our self-built medical text dataset is intentionally imbalanced to closely mirror the actual patient visit ratios across clinical departments. This design choice aims to simulate real-world application scenarios, thereby enabling more authentic evaluation of the model's discrimination capability between minority and majority class samples under natural data distributions.

In addition, a publicly available medical text dataset is employed for the purpose of validating the performance of the model under consideration in other medically relevant text categorization tasks, which can be accessed via https://huggingface.co/datasets/123rc/medical_text. This dataset encompasses the abstract portion of 14,500 medical literature, meticulously divided into training, validation, and test sets in a proportion of 8:1:1. The abstracts in the dataset vary in length, ranging from 140 to 2,000 characters. The abstracts are classified into five distinct disease categories: tumors, digestive disorders, neurological disorders, cardiovascular disorders, and general pathological conditions. The percentage of each category is displayed in the following Table 3.

**Table 3:** Percentage of each category in the medical literature abstract dataset.

**Label**	**Percentage of abstract dataset**
Neoplasms	21.9%
Digestive system diseases	10.3%
Nervous system diseases	13.3%
Cardiovascular diseases	21.1%
General pathological conditions	33.3%

The experimental environment of this paper is shown in Table 4.

**Table 4:** Settings of experimental environment.

**Name of experimental environment**	**Configurations**
Operating system	Windows 11
GPU	NVIDIA GeForce RTX4060ti
CPU	Intel Core i5-13490F
python	3.8
pytorch	1.12.1
Coding Format	Utf-8

### 4.2 Hyperparameter setting

The hyperparameter settings of the EDT-MCFEF model in the two Chinese datasets are presented in Table 5. In order to prevent the model from overfitting, the Early Stopping method was employed during the training process. In the event of an absence of improvement in the model's efficacy following 1,500 batch training iterations, the model will conclude the training process prematurely (β denotes the temperature parameter associated with the feature ensemble strategy outlined in Section 3.2).

**Table 5:** Settings of hyperparameter.

**Parameter name**	**Self-built medical datasets**	**Medical literature abstract dataset**
Hidden size	768	768
Learning rate	3e-5	3e-5
Batch size	128	128
Pad size	64	64
Optimizer	BERTAdam	BERTAdam
Epoch	15	20
Dropout	0.1	0.2
Num filters	(3, 4, 5)	(3, 4, 5)
β	0.5	0.3
Heads(MHA)	16	20
N-gram	3-gram	3-gram

### 4.3 Model comparison and analysis on self-built medical datasets

In this experiment, the evaluation metrics of the model will be the accuracy with weights (w-Acc) and the F1-score with weights (w-F1). In order to ascertain the superiority of the EDT-MCFEF model on the emergency department triage tasks, the EDT-MCFEF model is compared with the following 7 models. To ensure fair comparisons, all seven baseline models were trained and fine-tuned under identical conditions. On the self-built medical text dataset, we performed random search on each model using the validation set, exploring learning rates in the range [1e-5, 5e-5], batch sizes in {32, 64, 128}, and dropout rates in {0.1, 0.2, 0.3}. An early stopping strategy was applied—training was halted if the weighted F1 score showed no improvement over 1,500 consecutive batches–to prevent overfitting. Finally, for each model, the hyperparameter combination that achieved the highest weighted F1 on the validation set was selected as the final configuration for evaluation on the testing set:

- **BERT-base** (14): this model will use only the pre-trained BERT model to complete word vector processing, feature extraction, and classification.
- **BERT-TP** (22): this model utilizes a triage prioritization method to label diagnosis combinations in medical records and incorporates a composite loss function based on cost-sensitive learning into the BERT architecture before it is applied for word vector processing, feature extraction, and classification.
- **ERNIE 3.0** (18): this model utilizes the pre-training ERNIE model for word vector processing, feature extraction, and classification. ERNIE is a pre-training model that has been specifically designed for the processing of Chinese text.
- **RoBERTa-base** (15): this model will utilize the pre-trained model RoBERTa exclusively for word vector processing, feature extraction, and classification.
- **LLaMA-7B** (19): this model will utilize the pre-trained model LLaMA exclusively for word vector processing, feature extraction, and classification.
- **RoBERTa-LSTM-CNN (RB-LC)** (16): this model employs the pre-trained RoBERTa model for word vector processing and the LSTM-CNN model for feature extraction and classification.
- **RoBERTa-RCNN-Attention (RB-RA)** (22): this model utilizes a pre-trained RoBERTa model for word vector processing and incorporates an additional attention pooling layer after feature extraction using the RCNN (RNN-CNN) model to reduce feature dimensions and complete the classification outputs.

The results of the ablation experiments are shown in Table 6. Each model configuration was run independently three times, and the standard deviations were calculated. To evaluate the statistical significance of the performance differences, paired *t*-tests were conducted on the weighted F1 scores between each model configuration and the complete EDT-MCFEF model. Prior to applying the *t-*tests (p-t), we performed the Shapiro-Wilk normality test (p-sw) on the paired differences to verify the assumption of normality. The results indicated no significant deviation from a normal distribution (p-sw > 0.05), thereby justifying the use of the paired *t*-test for statistical analysis. The paired *t*-test is a classical statistical method widely used to evaluate the difference in mean performance between two models on the same test set over multiple runs. Its advantage lies in accounting for the correlation between paired samples, thereby increasing the test's statistical power. Also, the Shapiro-Wilk test, recommended for small sample sizes, was employed to ensure the validity of the normality assumption essential for the paired *t*-test.

**Table 6:** Experimental results of different models on Self-Built Medical Datasets, including paired *t*-test (p-t) and Shapiro-Wilk test (p-sw) *p-*values.

**Model**	**Self-built medical datasets**			
	w-Acc	w-F1	p-t	p-sw
BERT-base (2018)	0.8658 ± 0.0012	0.8635 ± 0.0010	0.007	0.537
BERT-TP (2023)	0.8720 ± 0.0010	0.8705 ± 0.0010	0.011	0.806
ERNIE 3.0 (2021)	0.8680 ± 0.0009	0.8669 ± 0.0008	0.008	0.253
RoBERTa-base (2019)	0.8703 ± 0.0007	0.8694 ± 0.0006	0.010	0.298
LLaMA-7B (2023)	0.8710 ± 0.0007	0.8701 ± 0.0008	0.010	0.637
RB-LC (2023)	0.8715 ± 0.0006	0.8679 ± 0.0004	0.009	0.843
RB-RA (2024)	0.8812 ± 0.0005	0.8829 ± 0.0004	0.013	0.463
**EDT-MCFEF (Ours)**	**0.8880** **±0.0002**	**0.8871** **±0.0003**	-	-

The bold values in the table indicate the results achieved by our model in the experiments.

As evidenced by the experimental results, the EDT-MCFEF model demonstrates superior performance in terms of accuracy and F1 value when compared to other models on the self-build medical dataset. The BERT model, which exhibits the poorest performance, with a 2.22% reduction in accuracy and a 2.36% reduction in F1 value in comparison to the EDT-MCFEF model. The medical dataset contains a greater number of categories and is characterized by a more colloquial style of text, as well as an imbalance in the categories. This renders the majority of models ineffective for section classification, with the sub-model classes being particularly susceptible to the influence of irrelevant semantic features. However, this also demonstrates the superior resilience of the EDT-MCFEF model in addressing the challenges posed by unbalanced and complex text. This result indicates that EDT-MCFEF can capture key information in medical texts more effectively through multi-channel feature extraction and dynamic fusion. The model performs particularly well in handling spoken and unbalanced emergency department triage data. In addition, the LLaMA model exhibits slightly lower performance than EDT-MCFEF on the medical dataset, with an accuracy of 87.40% and an F1 value of 87.31%. This finding suggests that, while LLaMA, as a large language model, possesses robust semantic comprehension capabilities, its static feature extraction method is unable to dynamically adjust the weights of global and local features in the emergency department triage task, as observed in the EDT-MCFEF model. This deficiency results in a marginally lower performance.

To more thoroughly validate whether the observed performance differences are statistically significant, we conducted three independent training and testing runs for each model on our self-built emergency triage dataset. This approach ensures the stability and comparability of the results. Based on these experiments, we performed paired *t*-tests between the weighted F1 scores of each baseline model and our proposed EDT-MCFEF model to assess the statistical significance of their performance differences. The experimental results indicate that even for RB-RA–the strongest baseline model with the smallest performance gap from EDT-MCFEF–the differences in performance across the three runs are still statistically significant (p-t < 0.05) when compared to EDT-MCFEF. This demonstrates that the performance improvement achieved by EDT-MCFEF is not only superior in absolute terms, but also unlikely to be the result of random variation. In summary, the superiority of EDT-MCFEF is consistent and reliable, further validating its effectiveness and practical value in the task of intelligent emergency department triage.

### 4.4 Model comparison and analysis on Medical Literature Abstract dataset

In addition to the validation on the self- built dataset, we also used the same 7 benchmark models on the Medical Literature Abstracts dataset to compare the performance with our model, and the results of the comparison experiments are shown in Table 7.

**Table 7:** Experimental results of different models on Medical Literature Abstract Dataset.

**Model**	**Medical literature abstract dataset**	
	**w-Acc**	**w-F1**
BERT-base (2018)	0.8003	0.7990
BERT-TP (2023)	07992	0.7982
ERNIE 3.0 (2021)	0.7999	0.7990
RoBERTa-base (2019)	0.8060	0.8051
LLaMA-7B (2023)	0.8265	0.8260
RB-LC (2023)	0.8183	0.8169
RB-RA (2024)	0.8224	0.8213
**EDT-MCFEF**	**0.8271**	**0.8258**

The bold values in the table indicate the results achieved by our model in the experiments.

Despite the presence of stylistic differences between the terminology employed in the medical literature and that utilized in the dictated texts of emergency department patients, the terminology used in the medical field in literature abstracts is characterized by heightened specialization and a greater text length. Consequently, the results of the comparison experiments remain informative. The findings reveal a substantial decline in the efficiency of the EDT-MCFEF and LLaMA models when evaluated against the self-built dataset. However, both models demonstrate superior performance compared to RB-LC and RB-RA. Notably, the EDT-MCFEF model exhibits an accuracy of 82.71%, marginally surpassing the LLaMA model. The LLaMA model's superiority is attributable to its increased capacity for parameters and more intricate configuration. The LLaMA model exhibits an aptitude for discerning more intricate linguistic patterns and domain-specific knowledge when confronted with literature summaries. In contrast, our model, EDT-MCFEF, meticulously considers the distinctive attributes of medical texts and the requisites of the classification task. EDT-MCFEF employs a hybrid masking strategy and a multi-channel feature extraction method, facilitating the model's capacity to assimilate text features in a more exhaustive manner. This enhancement is particularly pronounced in the comprehension of technical terms and extended sentences. Concurrently, our multi-channel feature fusion strategy enables the model to dynamically adjust the weights of individual channel features, thereby enhancing the accuracy and robustness of medical text classification.

A summary of the experimental results indicates that, in the emergency department triage task, EDT-MCFEF outperforms other models in most cases. This is due to three factors: a hybrid mask strategy in the feature augmentation layer, a multi-channel feature extraction strategy in the feature extraction layer, and a multi-channel feature fusion strategy in the ensemble learning layer. These strategies allow for the extraction of richer and more comprehensive semantic information from text while reducing the interference of irrelevant semantic information. This results in better performance and robustness.Unlike conventional single-channel pre-trained models (RoBERTa-base, ERNIE 3.0), EDT-MCFEF employs a multi-channel structure that parallelizes Multi-Head Attention (MHA) for global dependency extraction and CNN for local key-phrase capture. This is further enhanced by a temperature-modulated dynamic fusion mechanism at the feature level, which adaptively adjusts weights based on the semantic characteristics of input texts. This design demonstrates superior feature discrimination capability, particularly in handling medical long-text scenarios where balancing contextual information and critical symptom descriptions is essential; Compared to fusion models like RB-RA, our approach avoids fixed concatenation or attention pooling. Instead, it leverages a softly controlled probabilistic weight fusion mechanism to dynamically adjust the contribution of each channel. This significantly improves the model's context-aware adaptability, enabling more nuanced handling of diverse medical text patterns; While large-scale models like LLaMA-7B exhibit stronger generalization due to their vast parameters, their static feature processing lacks task-specific weight optimization. In practice, this leads to marginally inferior performance despite their theoretical advantages. Our method's adaptive mechanism proves more effective in targeted clinical text analysis tasks.

### 4.5 Case study

In order to further analyze the advantages of the EDT-MCFEF model in comparison to LLaMA, RB-LC, RB-RA, this paper employs the aforementioned four trained models to predict the oral text samples of emergency patients for analysis. Furthermore, this paper validates the emergency department triage performance of the EDT-MCFEF model. In this paper, the “Case Study” is not presented as a formal experiment or quasi-experiment, but rather as an explanatory example analysis aimed at intuitively demonstrating the performance differences among models when processing specific clinical expressions. By selecting representative patient input samples and comparing the outputs of different models, we highlight the advantages of EDT-MCFEF in capturing both global semantics and local clinical keywords. This section serves as a complement to the quantitative experiments, providing insight into the model's behavior in real-world triage contexts.

- Sample 1: My child has been coughing for the past two days, with thick yellow snot coming out of his nose and his temperature fluctuating around 37.3 degrees. What is the cause?

As illustrated in Table 8, EDT-MCFEF employs a dynamic feature fusion mechanism, integrating local symptoms such as “cough” and “thick yellow snot” with global age information, represented by the term “child.” This integration enables the model to accurately identify pediatric features and avoid the misjudgment inherent in other models that prioritize respiratory symptoms. Its inference speed (1.5 seconds) is 25% faster than that of LLaMA, which fulfills the real-time requirements of emergency departments.

- Sample 2: I have been feeling uncomfortable in my throat for the last week, I get sore, dry, itchy throat, more pain when swallowing, a mild cough, no phlegm or a small amount of white sticky phlegm. It's more noticeable when I'm in class, and it's better when I rest. I don't smoke either, but maybe drink occasionally.

**Table 8:** Inference and result analysis of each model for Sample 1.

**Model**	**Triage result**	**Is it correct?**	**Inference time**	**Key issues**
LLaMA	Pulmonology	×	2.0s	The analysis overlooks “child” while overemphasizing respiratory symptoms like “coughing” and “thick yellow snot.”
RB-LC	Pulmonology	×	1.9s	The analysis neglects to integrate localized symptoms (“thick yellow snot”) with global factors like age information.
RB-RA	Pulmonology	×	1.9s	The attention mechanisms failed to suppress redundant features, particularly evident in how the “37.3°C” feature disrupted department categorization.
**EDT-MCFEF**	**Pediatrics**	✓	**1.5s**	**Hybrid masking enhances “child” feature recognition by dynamically integrating MHA (global age patterns) and CNN (local symptom detection) weight matrices**.

The bold values in the table indicate the results achieved by our model in the experiments.

As demonstrated in Table 9, EDT-MCFEF effectively differentiates between the primary symptom (feeling uncomfortable in my throat) and the secondary symptom (cough) by leveraging the synergistic effect of multi-channel features. This approach circumvents the misdiagnosis that arises from feature confusion in other models. Its inference speed (1.9 seconds) is 17.4% faster than that of LLaMA, thereby enhancing efficiency while maintaining accuracy.

**Table 9:** Inference and result analysis of each model for Sample 2.

**Model**	**Triage result**	**Is it correct?**	**Inference time**	**Key issues**
LLaMA	Pulmonology	×	2.3s	The misdiagnosis stems from misclassifying “cough” and “white sticky phlegm” as respiratory conditions while neglecting throat-specific indicators like “sore throat” and “painful swallowing.”
RB-LC	Pulmonology	×	2.3s	Inadequate extraction of localized features, failing to distinguish the primary relationship between “sore throat” and “cough.”
RB-RA	Otolaryngology	✓	2.2s	The keyword “sore throat” is captured by attention, but reasoning is slow.
**EDT-MCFEF**	**Otolaryngology**	✓	**1.9s**	**The CNN architecture enhances localized symptom features (“sore throat,” “painful swallowing”) while MHA contextualizes global patterns ("relief during rest”), with dynamic weighting significantly suppressing cough interference**.

The bold values in the table indicate the results achieved by our model in the experiments.

In summary, the EDT-MCFEF model demonstrates the significant advantage of multi-channel feature co-innovation in emergency department triage. The fundamental breakthrough lies in the construction of a bi-directional complementary feature extraction system: on the one hand, the global contextual dependencies (e.g., the correlation between the age attribute of "child" and "temperature fluctuation" in Case 1 are captured by the MHA, and on the other hand, the keyword features of the local symptoms are extracted with the help of Multi-scale CNN (e.g., the n-gram features such as "sore throat" and "swallowing pain" in Case 2). The model's capacity to adjust the contribution of global semantics and local features is enhanced by the incorporation of a dynamic weight fusion module with the temperature parameter β. Concurrently, the hybrid masking strategy serves to augment features using a two-pronged approach. The n-gram dynamic mask enhances local symptom representations (e.g., "thick yellow snot") through a sliding window, while the filler mask retains the key context (e.g., the course of the disease feature of "remission after rest" in Case 2). Due to the aforementioned advantages, the model can achieve real-time reasoning for emergency department triage scenarios in 1.5-2.0 seconds for a single sample, which is more than 25% faster than the traditional model. This innovative integration of multi-channel feature fusion and structural augmentation offers an intelligent solution for emergency department triage, achieving a balance between medical logic and computational efficiency.

### 4.6 The ablation experiment and analysis of modules

The objective of this study is to validate the impact of each module in the EDT-MCFEF model on the emergency department triage task and to gain insight into the performance of each module of the model in both unbalanced and balanced datasets. To this end, the following ablation experiments have been designed (w/o stands for “without”):

- w/o hybrid mask: this designation signifies the hybrid mask strategy in the EDT-MCFEF model, excluding the feature augmentation layer.
- w/o TextCNN: denotes the local semantic features extracted by TextCNN in the EDT-MCFEF model, excluding the feature extraction layer.
- w/o MHA: denotes the global semantic features extracted by MHA in the EDT-MCFEF model, minus the feature extraction layer.
- w/o multi-channel feature fusion: denotes the direct output of the fully connected layer, which is obtained without employing the multi-channel feature fusion strategy of the ensemble learning layer in the EDT-MCFEF model. This is achieved after the global and local semantic features have been extracted.
- w/o temperature parameter: this indicates that the temperature parameter β is not employed in the multi-channel feature integration strategy of the ensemble learning layer in the EDT-MCFEF model after the extraction of the global and local semantic features.

The results of the ablation experiments, each conducted over three independent runs per model configuration with standard deviations computed, are presented in Table 7. Additionally, paired *t*-tests were performed on the weighted F1 scores (w-F1) between each model configuration and the complete EDT-MCFEF model to assess the statistical significance of the performance differences. Prior to applying the *t*-tests (p-t), we conducted the Shapiro-Wilk normality test(p-sw) on the paired differences to verify the assumption of normality. The results showed no significant deviation from a normal distribution (p-sw > 0.05), supporting the validity of the paired *t*-test.

As evidenced by the experimental results, the hybrid mask-based feature augmentation, multi-channel feature extraction, and multi-channel feature fusion strategy based on ensemble learning in the EDT-MCFEF model have a pronounced impact on the performance enhancement of the model. In comparable circumstances, the multi-channel feature extraction and multi-channel feature fusion strategies have a more pronounced beneficial impact on the model. This suggests that the approach of extracting global semantic information and local semantic information and integrating and fusing the two can effectively assist the model in accurately producing classification results on the classification task. The removal of the feature augmentation based on the hybrid mask strategy from the EDT-MCFEF model resulted in a discernible decline in its performance on the two datasets. This observation substantiates the assertion that the hybrid mask plays a pivotal role in feature augmentation for unbalanced datasets.

As shown in the ablation results in Table 10, each module in the EDT-MCFEF model contributes to performance improvement, albeit with seemingly modest numerical differences. However, such differences—ranging from 0.4% to 0.9% in weighted F1—are non-trivial in the context of modern NLP tasks, especially in medical text classification where most baseline models already operate near performance saturation. For example, the removal of the hybrid mask-based feature augmentation results in a relatively smaller performance drop (from 0.8871 to 0.8791, p-t = 0.010), but this still highlights the mask's role in improving model generalization under unbalanced data conditions. The hybrid mask helps the model focus on diverse and non-redundant features, which is critical when certain class patterns are underrepresented. This is particularly important in medical text classification, where medical data often exhibit class imbalance, with certain diseases or symptoms being significantly less represented than others.

**Table 10:** Results of ablation experiments of EDT-MCFEF on medical datasets, including paired *t*-test (p-t) and Shapiro-Wilk test (p-sw) p-values.

**Model**	**Medical datasets**			
	**w-Acc**	**w-F1**	**p-t**	**p-sw**
w/o hybrid mask	0.8810 ± 0.0006	0.8791 ± 0.0005	0.010	0.899
w/o TextCNN	0.8810 ± 0.0005	0.8812 ± 0.0004	0.011	0.780
w/o MHA	0.8785 ± 0.0007	0.8785 ± 0.0006	0.010	0.637
w/o multi-channel feature fusion	0.8829 ± 0.0004	0.8812 ± 0.0005	0.012	0.832
w/o temperature parameter	0.8848 ± 0.0003	0.8835 ± 0.0004	0.015	0.363
**EDT-MCFEF**	**0.8880** **±0.0002**	**0.8871** **±0.0003**	-	-

The bold values in the table indicate the results achieved by our model in the experiments.

Among the three core components, the multi-channel feature extraction and dynamic fusion strategy exhibit the most consistent impact. Their integration enables the model to simultaneously capture global contextual dependencies and local discriminative expressions, leading to more accurate and stable classification results. The temperature-controlled fusion mechanism further enhances this effect by adaptively balancing the contributions of each feature channel based on the input characteristics. In summary, although no single component leads to a dramatic performance drop, the complete configuration of EDT-MCFEF achieves the best performance. This confirms the complementary nature of its modules and justifies their inclusion as an integrated design to enhance robustness and generalization.

### 4.7 Experimental analysis of ablation with temperature parameter β

**Figure 9:**
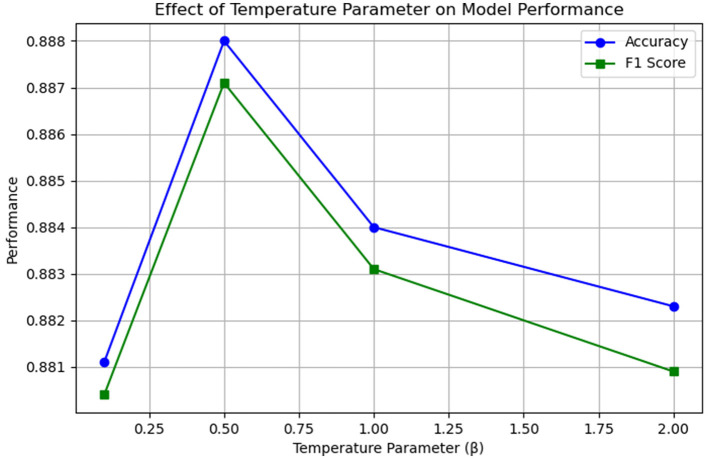
Results of ablation experiments with temperature parameter β.

The objective of this study is to ascertain the impact of the temperature parameter in the multi-channel feature fusion strategy employed in the EDT-MCFEF model on the emergency department triage task. Additionally, the aim is to gain insight into how the model learns to identify and extract the comprehensive features present in the text. To this end, the study employs a series of β parameters for experimental comparison and trains them on a medical dataset. This approach is undertaken to identify the optimal temperature value, which will facilitate more effective control over the distribution of fusion weights. Figure 9 below illustrates the impact of temperature values on the model.

The results of this ablation experiment demonstrate that the temperature parameter has a measurable and consistent impact on the model's feature fusion behavior and performance. When β is relatively small (β < 0.5), the distribution of the output of the multi-channel feature fusion strategy is sharper, which may result in an over-reliance of the model on a single feature (MHA or CNN). Conversely, when β is relatively large (β > 0.5), the distribution of the output of the fusion strategy is smoother, but this may lead to a lack of focus on important features. It can be observed that when a moderate temperature parameter (β = 0.5) is established, the model is capable of effectively balancing global and local feature information, thereby enhancing its overall performance.

### 4.8 Analysis of confusion matrices

**Figure 10:**
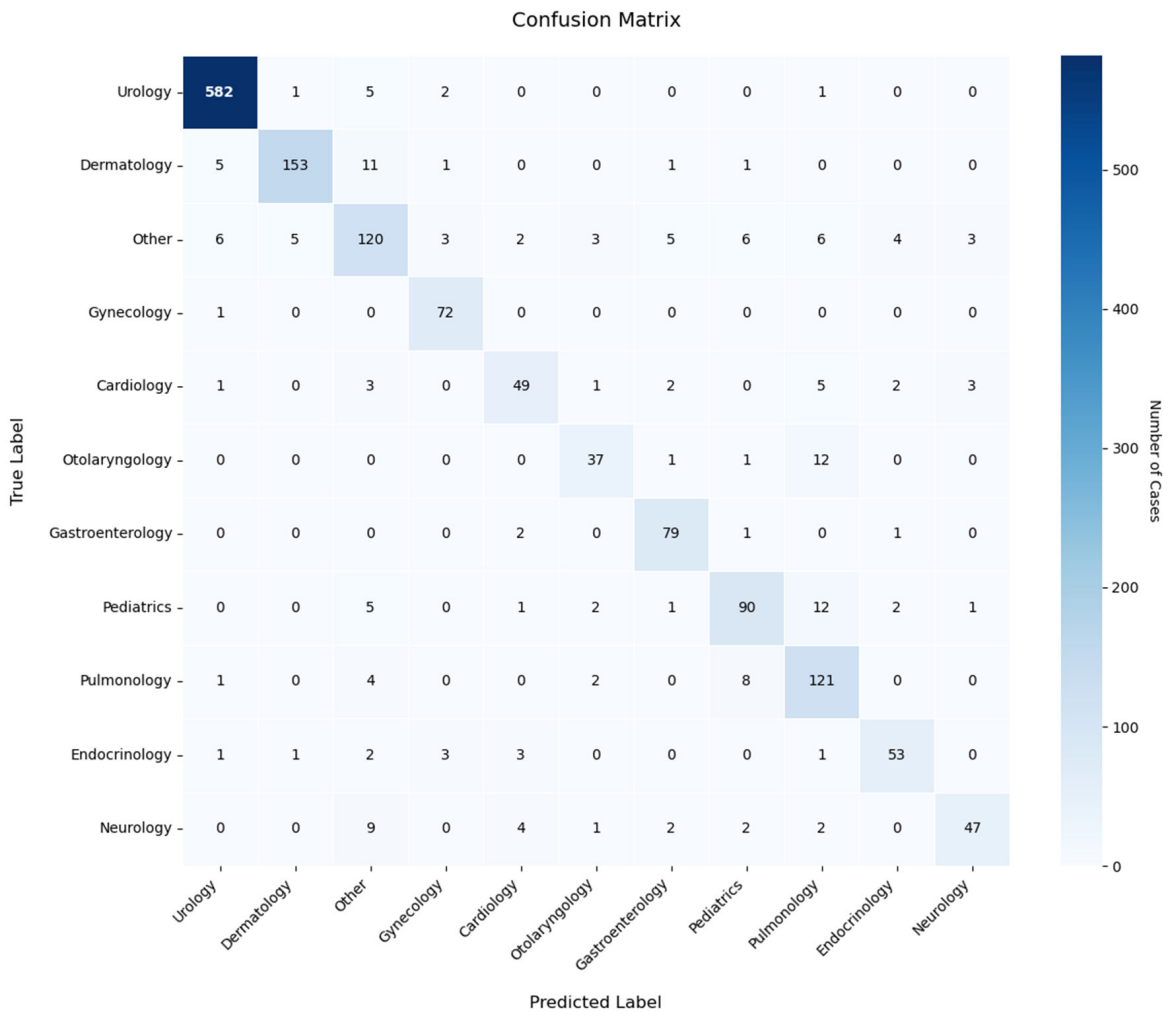
Confusion matrix of the model in the emergency department triage task.

To gain a more detailed understanding of the classification performance of the model, we conducted an analysis using a confusion matrix. A confusion matrix is a tool used to assess the performance of a classification model. It provides a visual representation of the model's classification outcomes, including the number of correctly and incorrectly classified instances. As illustrated in Figure 10, the confusion matrix results for the EDT-MCFEF model demonstrate an 88.80% accuracy rate in emergency department triage tasks.

Analysis of the confusion matrix reveals distinct performance patterns across medical specialties. The model demonstrates robust classification capabilities for urology texts (582/591 correct classifications), dermatology, and gynecology categories, indicating effective extraction of discriminative sequence features. However, systematic misclassification emerges in two critical aspects: (1) The “Other” category exhibits bidirectional classification errors, with both frequent misassignments to this category and improper allocations from it, suggesting inadequate handling of low-information samples. (2) Cross-department confusion persists, notably between otolaryngology-pulmonology (12/51 cases) and pediatrics-pulmonology (12 cases), revealing sensitivity to overlapping symptom descriptions (e.g., respiratory-related features). These patterns imply two underlying limitations: first, the model's decision thresholding requires optimization for cases with ambiguous feature representations, as evidenced by its propensity for definitive classification rather than conservative “Other” assignment. Second, inter-departmental feature overlap—particularly in symptoms spanning multiple specialties—challenges the current feature differentiation framework. The error distribution suggests targeted dataset augmentation could yield performance gains, specifically through expanded exemplars of boundary cases and improved annotation granularity for polysemous clinical descriptors. These findings underscore the necessity for dynamic weighting mechanisms that better account for diagnostic hierarchy and symptom specificity in medical text classification tasks.

## 5 Conclusion

As a fundamental technique in natural language processing (NLP), text classification demonstrates extensive applicability across multiple domains, with established implementations in information retrieval systems (28), news taxonomy frameworks (29), and sentiment analysis architectures (30). This methodology enables systematic organization of unstructured textual data through automated categorization mechanisms, serving as critical infrastructure for contemporary data-driven decision systems. The operational effectiveness stems from its capacity to transform raw text inputs into structured semantic representations, thereby facilitating efficient knowledge management and pattern recognition across heterogeneous information spaces.

This paper addresses two issues about the daily triage of patients in emergency departments. The objective is twofold: firstly, to develop an efficient method for processing and analyzing a substantial volume of patient medical record data; and secondly, to identify a means of extracting pertinent information from unstructured spoken text, to assist triage decision-making. To achieve this, the EDT-MCFEF model has been proposed as a potential solution. The model's objective is to mitigate the impact of irrelevant and redundant semantic information on emergency department triage, enhance the extraction and incorporation of key semantic information from text, and propose a hybrid masking strategy, multi-channel feature extraction, and ensemble learning with multi-channel feature fusion to address the aforementioned issues.

As demonstrated in our experiments, the experimental findings indicate that the emergency department triage model, founded upon multi-channel feature extraction and fusion, exhibits superior performance in comparison to other benchmark models on both the imbalanced medical dataset and the balanced THUCNews dataset. This observation underscores the model's enhanced generalization capability and robustness. The results imply that the model proposed in this paper is more adept at circumventing the impact of extraneous information and efficaciously extracting richer semantic features. Furthermore, the analysis of both the example and the confusion matrix demonstrates that the EDT-MCFEF model enhances the model's inference speed while maintaining high accuracy, thereby underscoring the model's enhanced practicability and generalizability.

The EDT-MCFEF model proposed in this study has the potential to transform medical practice in two distinct ways. Firstly, it reduces the reliance on manual experience, thereby reducing the decision-making burden of emergency medical personnel in high-stress environments and reducing the risk of delays due to subjective misjudgments. Concurrently, the real-time reasoning speed of 1.5–2 seconds is considerably superior to that of the conventional manual triage process, thereby enabling the prioritization of patients with acute and serious illnesses for treatment. Furthermore, the model's modular architecture exhibits cross-domain extensibility, which can be employed in outpatient pre-screening and intelligent categorization of medical literature by adjusting the training dataset. Future research could further apply this model to the field of mental health screening, such as identifying language related to anxiety or depression. By leveraging the multi-channel architecture to separately extract emotional context and local keywords, the model has the potential to enhance the automation and accuracy of early intervention. Additionally, it can be migrated to non-medical scenarios, such as grading customer service work orders in financial services and annotating the types of legal cases.

### 5.1 Threats to external validity and conclusion validity

Despite the model's enhancements in performance, as outlined in this paper, several areas necessitate attention. Currently, the model has been primarily evaluated on Chinese medical texts, a self-built emergency triage dataset, and medical literature abstracts. Due to the limited data sources, the model may face certain adaptability challenges in cross-lingual and cross-institutional scenarios. The model's training time and parameter count are expected to rise due to its intricate structure and the laborious nature of feature extraction. As demonstrated in the confusion matrix graph in this paper, the model has met the standard of triage in terms of overall accuracy. However, the method demonstrates persisting limitations in handling multi-system symptom crossover scenarios, particularly regarding inter-specialty diagnostic ambiguity such as cardiology-neurology misclassification risks, which warrants further refinement for clinical deployment.

Although we conducted three independent runs and applied paired *t*-tests with Shapiro–Wilk normality verification, it should be emphasized that the sample size remains relatively small. As a result, the statistical power of the paired *t*-test is limited, and the resulting p-values should be interpreted as preliminary evidence rather than definitive confirmation of significant differences. Power analysis was not conducted in this study due to the small sample size. In future work, we plan to conduct more comprehensive experiments with increased sample size and include statistical power analysis to better support the robustness of significance testing.

### 5.2 Future work

Although the model proposed in this paper demonstrates incremental improvements in performance, it is important to note that due to the complexity of the model structure and the additional intricacies introduced by the feature extraction steps, the running time and overall number of parameters may increase, resulting in higher computational costs. Therefore, we conducted normality verification and paired *t*-tests on the results of three independent experiments to evaluate the statistical significance of performance differences. Moving forward, we plan to perform more comprehensive statistical validation on multiple public medical text datasets using high-performance computing resources. This includes applying methods such as ANOVA and Kruskal-Wallis tests to systematically evaluate the robustness and significance of performance differences across varying tasks and data characteristics. These efforts will further enhance the scientific validity and credibility of the model's evaluation and support its broader application in clinical practice.Furthermore, the experimental development process in this study lacks a fully explicit separation of planning, execution, and data collection phases. This may hinder the reproducibility of our approach, as recommended in reproducible AI research standards. Future work will aim to modularize these stages to improve transparency and facilitate replication.

Further research could be conducted in the following areas: the RoBERTa architecture can be optimized by the characteristics of medical text. Pre-training can be conducted using a substantial quantity of pertinent data, thereby enabling the RoBERTa model to become more specialized in the context of emergency department triage. Furthermore, the computational cost of the model can be reduced and its actual deployment efficiency can be enhanced through the implementation of more efficient model compression and acceleration techniques. Secondly, in conjunction with practical implementation, future research may entail further integration of multimodal data, such as the combination of electronic medical records (EMR) and patient voice data, to develop a more comprehensive emergency department triage system. This approach could enhance overall diagnostic accuracy and efficiency.

## Data Availability

The raw data supporting the conclusions of this article will be made available by the authors, without undue reservation.
